# MicroMundo Upside Down: Targeted Searching for Antibiotics-Producing Bacteria From Soil With Reverse Antibiosis Approaches

**DOI:** 10.3389/fmicb.2020.577550

**Published:** 2020-10-15

**Authors:** María Alvarado, Pilar Clemente-Casares, Diego A. Moreno, Piet W. J. de Groot

**Affiliations:** ^1^Facultad de Farmacia, Universidad de Castilla-La Mancha, Albacete, Spain; ^2^Centro Regional de Investigaciones Biomédicas, Universidad de Castilla-La Mancha, Albacete, Spain; ^3^Universidad Politécnica de Madrid, Escuela Técnica Superior de Ingenieros Industriales (ETSII-UPM), Madrid, Spain; ^4^Facultad de Medicina, Universidad de Castilla-La Mancha, Albacete, Spain; ^5^Fundación Parque Científico y Tecnológico de Castilla-La Mancha, Albacete, Spain

**Keywords:** antibiotics, antimicrobial resistance, actinomycetes, ESKAPE pathogens, Tiny Earth, MicroMundo, service-learning

## Abstract

Tiny Earth (TE) is a popular international citizen science program aimed at improving public awareness on the growing antimicrobial resistance problem of which MicroMundo Albacete is a Spanish node. With a protocol that is focused on the isolation of antibiotics-producing actinomycetes from soil, 70% of the high school students in MicroMundo Albacete 2020 isolated colonies with antagonistic activity against Gram-positive tester bacteria. However, no activity was found against Gram-negative bacteria. Here, we further adapted the protocol toward a more targeted screening that also enables isolation of antagonistic bacteria against Gram negatives using two different reverse-antibiosis approaches involving a spraying technique or flipping soil sample disks upside down. Exploiting the soil samples from MicroMundo Albacete 2020, the new approaches yielded isolation of actinomycete bacteria with antagonistic activity against Gram-negative as well as Gram-positive tester bacteria. We propose that (educational) science programs which aim to search for antibiotic-producing bacteria may implement these approaches in their protocol to promote a successful and stimulating outcome of the experiment for the participating students.

## Introduction

Continued emergence of multidrug-resistant bacteria is considered a growing and global human health threat. The number of new antibiotics that have been launched in recent years, especially those with activity against Gram-negative bacteria, is very limited and seems insufficient to combat the so-called “antibiotic crisis” (Wright, 2015; Butler et al., 2017; Van Puyvelde et al., 2018). As a consequence, toward the second half of the 21st century, this may bring our vulnerability for acquiring lethal microbial infections back to the situation how it was before the discovery of antibiotics (Leung et al., 2011; Laxminarayan et al., 2013; O’Neill, 2016). On the positive side, governmental regulations on the use of antibiotics in many developed countries have recently become stricter and have forbidden their application in poultry feed.

Nevertheless, crucial to prevent a devastating future perspective is to achieve a better public awareness on the proper use antimicrobials. Tiny Earth (TE) is a very successful and popular crowdsourcing (also named studentsourcing or citizen science) program that is implemented worldwide to address the antimicrobial drug resistance (AMR) issue (Hernandez et al., 2018). The basic idea of the program is to enhance awareness on the AMR problem among students by letting them participate in a real and exciting experiment that is aimed at antibiotic discovery. In Spain, the program, named MicroMundo, is run in a service-learning set-up in which undergraduate students, after receiving training by academic researchers, perform as teaching assistants during teaching sessions with high school classes (Valderrama et al., 2018; De Groot et al., 2019). When running the program for the first time at the University of Castilla-La Mancha (UCLM) in Albacete in 2019, we decided to focus the experimental protocol on isolation of Gram-positive actinomycetes, the main producers of most clinically used antibiotics (Procópio et al., 2012; Barka et al., 2016; Butler et al., 2017). Doing so, a higher percentage of the isolated bacteria with antagonistic activity against tester bacteria was obtained, making the project more alluring for the participating students (De Groot et al., 2019). However, with this strategy, it appears more likely to pick up antagonistic activity against Gram-positive than against Gram-negative bacteria as all of the positive isolates produced antagonistic activity against Gram positives but no activity against Gram-negative tester bacteria was observed (De Groot et al., 2019).

To implement a more targeted screening that will make the protocol more amenable to also pick up activity against Gram-negative bacteria, we, here, designed a further modification to the protocol, functionality of which is demonstrated with soil samples from the MicroMundo 2020 project.

## Materials and Methods

### MicroMundo Albacete 2020

The set-up including methodological details of the MicroMundo Albacete service-learning project is described in De Groot et al. (2019). In 2020, the program was run at two high schools [IES Tomás Navarro Tomás (TNT) and IES Andrés de Vandelvira (AV)]. Twenty student pairs each analyzed 10 colonies from self-taken soil samples (taken in the provinces of Albacete and Cuenca) using two different growth media [Reasoner’s 2A agar (R2A, Oxoid) in TNT and Actinomycete Isolation Agar (AIA, Sigma-Aldrich) in AV] that favor growth of actinomycetes. Both media were supplemented with the antimicrobials dieldrin (4 mg/L), nalidixic acid (20 mg/L), and cycloheximide (80 mg/L) to inhibit growth of mites, Gram-negative bacteria, and fungi, respectively. The final antibiosis experiment of the purified bacterial isolates was performed with Gram-positive tester bacteria *Bacillus subtilis* (strain ATCC 6051) and *Staphylococcus epidermidis* (ATCC 14990), and Gram-negative *Escherichia coli* (ATCC 11775). The experiment was repeated by experienced UCLM researchers to validate the antagonistic activity.

### Reverse Antibiosis Approaches

Aiming to achieve a more targeted screening of soil samples, two alternative reverse antibiosis approaches were developed: (i) plates grown with serial dilutions of soil samples, containing up to a few hundred colonies, were sprayed with 0.4–0.5 ml of ESKAPE-relative tester bacteria culture, freshly grown overnight in rich medium [LB Broth (Fisher Scientific) or Tryptic Soy Broth (TSB, Oxoid) at 30°C], using a spray bottle. (ii) Fully grown (10 days) soil sample agar plates were loosened from their Petri dishes and placed upside down in the lid of the dish. The bottom side now facing up was inoculated with a tester bacterium by painting the entire surface of the plate using a sterile cotton swab (Deltalab). In both methods, appearance of halos is monitored after 1–3 days of incubation at 30°C. Candidate bacterial colonies were purified by re-streaking on R2A or AIA plates with and without nalidixic acid, and their potential antagonistic activity was verified using the same antibiosis technique as in the MicroMundo project (De Groot et al., 2019) but using the original media (R2A and AIA) instead of TSB agar (TSA).

### Molecular Identification

The methodology for genomic DNA isolation of purified bacteria is described in De Groot et al. (2019). Amplification of the complete 16S rDNA gene was achieved using oligonucleotides fD1: 5'-AGAGTTTGATCCTGGCTCAG-3' and Rp2: 5'-ACGGCTACCTTGTTACGACTT-3' (Weisburg et al., 1991), purchased from STAB-Vida. PCR was performed using a proofreading KAPA HiFi PCR kit (KAPA Biosystems) following the manufacturer’s instructions. PCR fragments were checked on agarose gel, cleaned up (ExtractMe DNA clean-up kit, BLIRT), and sequenced (STAB-Vida). Primers 16S_339_F and 16S_1087_R (De Groot et al., 2019), were also used for sequencing. Taxonomic and phylogenetic DNA sequence analysis was performed using NCBI-Blast and MEGA X (Kumar et al., 2018) software.

## Results and Discussion

In the MicroMundo Albacete 2020 project, 200 selected bacterial isolates were tested for antagonistic activity against ESKAPE-like tester bacteria by high school students. The acronym ESKAPE refers to six leading nosocomial pathogens (*Enterococcus faecium, Staphylococcus aureus, Klebsiella pneumoniae, Acinetobacter baumannii, Pseudomonas aeruginosa,* and *Enterobacter* species) that exhibit multidrug resistance and virulence (Mulani et al., 2019). For biosafety reasons, in the studentsourcing program, these pathogenic bacteria are replaced by harmless “relatives.” Guided by results of the previous year (De Groot et al., 2019), we tested antagonistic activity against the Gram-positive ESKAPE-relatives *B. subtilis* and *S. epidermidis*, and against Gram-negative *E. coli*. Twenty-five of the isolates (12.5%) showed antagonistic activity against the Gram-positive *B. subtilis* and *S. epidermidis* (Supplementary Table S1), whereas no growth-inhibiting activity was detected against Gram-negative *E. coli*. Fourteen of the 20 (70%) student pairs detected at least one positive isolate in their soil samples. To demonstrate that positive isolates are (mostly) actinomycetes, 16S rDNA of isolates from 13 different soil samples was sequenced, in each case revealing highest identity levels with different *Streptomyces* species (Supplementary Table S1). This is not an unexpected result. Like many other *Streptomyces* spp. (Bagley et al., 2005), several of them are already known as producers of different types of antibiotics (Supplementary Table S1), and most antibiotics obtained from actinomycetes originate from *Streptomyces* spp. (Berdy, 2005; Barka et al., 2016). We consider the results of MicroMundo Albacete 2020 very positive and consistent with those of the previous year (De Groot et al., 2019). Once again it demonstrated that with a protocol directed toward isolation of actinomycetes, the majority of the high school students were able to isolate antibiotic-producing bacteria from soil.

However, as in the previous year, antagonistic activity against Gram-negative bacteria was not discovered during MicroMundo Albacete 2020, and this seems an undesired limitation of the chosen approach. Further, the MicroMundo project highly relies on serendipity as a limited number of actinomycete-like colonies (often 10) from a given soil sample are more or less randomly chosen for further purification and antibiosis tests. Aiming to improve the protocol on both aspects, we decided to exploit the soil samples of the MicroMundo project to develop two different reverse antibiosis approaches.

### Approach 1: Spraying Technique

**Figure 1:**
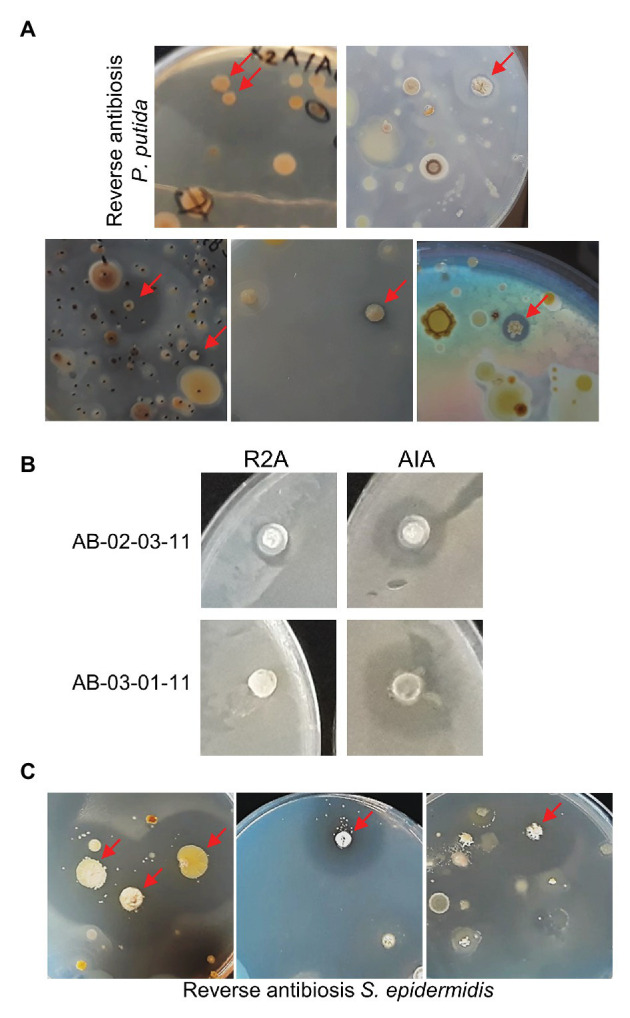
Reverse antibiosis spraying technique. **(A)** Examples of soil sample plates sprayed with the Gram-negative bacterium *Pseudomonas putida*. Colonies surrounded by halos are indicated by arrows. **(B)** Confirmative *P. putida* antibiosis testing of two purified positive colonies on R2A and Actinomycete Isolation Agar (AIA). **(C)** Examples of soil sample plates sprayed with the Gram-positive bacterium *Staphylococcus epidermidis*.

Some of the plates from which the students had selected their candidate colonies were carefully sprayed with an aliquot of fresh cultures of ESKAPE-relative tester bacteria. Prior growth tests indicated that *Pseudomonas putida* is the only Gram-negative ESKAPE-relative that can grow on media containing 20 μg/ml nalidixic acid (as present in the soil sample plates). After spraying *P. putida* and allowing time for growth, on about 25% of the soil sample plates containing up to a few hundred colonies, one or two colonies were apparently surrounded by small, but sometimes larger, halos (Figure 1A).

The spraying technique subsequently requires thorough purification of positive bacteria by re-streaking to eliminate the contaminating tester bacteria. Purification of positive candidates is in progress. Antibiosis testing of three already purified bacteria on the original R2A or AIA medium (without antibiotics) confirmed antagonistic activity of two of these isolates against *P. putida* (Figure 1B). Interestingly, both bacteria also showed slight antagonistic activity against other, Gram-positive and Gram-negative, ESKAPE-relative tester bacteria (Table 1). DNA sequencing revealed that both positive isolates are *Streptomyces* spp. (Table 1). Thus, the spraying technique allows for targeted screening of actinomycetes with antagonistic activity against Gram-negative bacteria in soil samples.

**Table 1:** Isolated antibiotic-producing bacteria using reverse antibiosis approach with *P. putida*.

Soil sample code	Growth medium	Size of halo (mm)^a^				NCBI-Blast result
		Bs^b^	Se	Pp	Ec	
		R2A/AIA	R2A/AIA	R2A/AIA	R2A/AIA	
AB-02-03-11	R2A + NA^b^	11/8	nh^a^/nh	8/13	nh/9	99.0% id. with *S. vastus* NBRC 13094
AB-02-03-12	R2A + NA	No antagonistic activity in antibiosis tests				
AB-03-01-11	AIA + NA	9/7	nh/10	9/17	nh/nh	99.9% id. with *S. gardneri* B547

a Size of agar disk is 6 mm. nh, no halo.

b Bs, *B. subtilis*; Se, *S. epidermidis*; Pp, *P. putida*; Ec, *E. coli*; NA, nalidixic acid.

In soil samples plated on R2A or AIA medium, colonies with antagonistic activity against Gram-positive bacteria are often abundant and even by random selection, it proves relatively easy for the high school students to find some positives. However, if one applies other growth conditions, this may be more challenging. Therefore, targeted screening using the spraying technique was also attempted using *S. epidermidis*, the only Gram-positive ESKAPE-relative that can grow, albeit very slowly, on R2A and AIA in the presence of 20 μg/ml nalidixic acid, as tester bacterium. Some soil samples that were shown to contain positive isolates in the MicroMundo project were re-plated on R2A or AIA media and sprayed with *S. epidermidis*. As expected, colonies surrounded by clear halos were observed already on plates containing only few colonies (Figure 1C). We conclude that the reverse antibiosis spraying method allows for a targeted screening of bacteria with antagonistic activity against Gram-positive as well as Gram-negative bacteria.

### Approach 2: Flipping Technique

**Figure 2:**
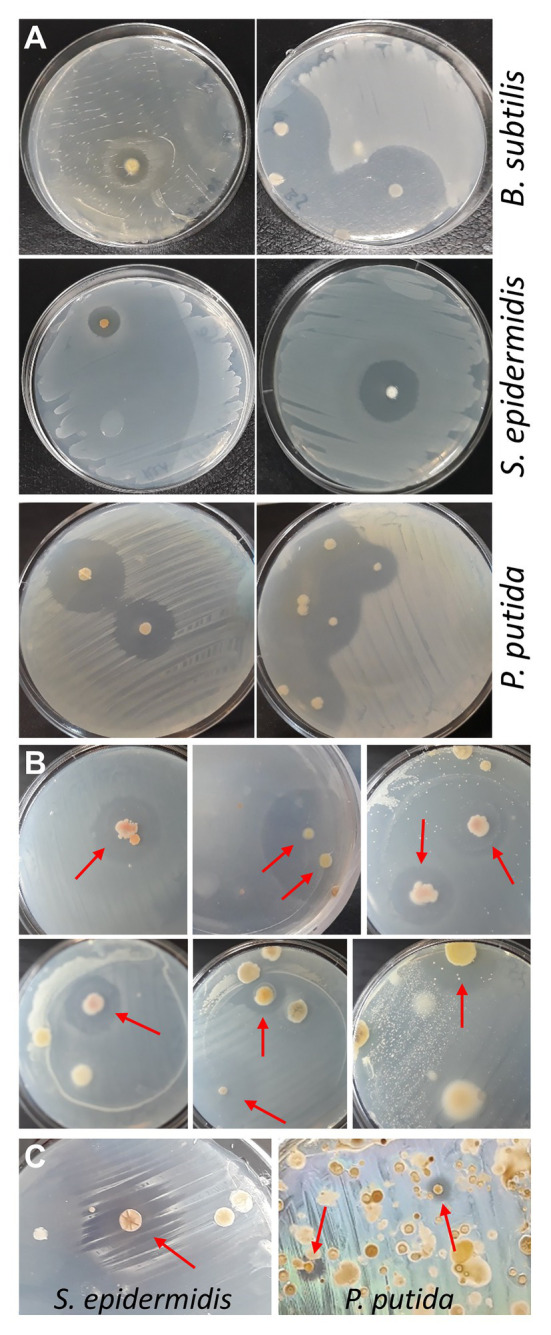
Reverse antibiosis flipping technique. **(A)** Testing of reverse-antibiosis flipping method using plates with purified positive actinobacteria. After flipping the agar upside down, plates were seeded with tester bacteria. Plates with purified positive actinobacteria from the MicroMundo 2020 project were seeded with *Bacillus subtilis* or *S. epidermidis*. Plates with purified actinobacteria that were identified to have activity against *P. putida* by the spraying method were seeded with *P. putida* (left, AB-02-03-11; right, AB-03-01-11). **(B)** Cultivated soil samples at dilutions 10^−6^ were flipped and seeded with the Gram-positive bacterium *S. epidermidis*. Colonies surrounded by halos are indicated by arrows. **(C)** Heat-treated soil sample AB-03-08, plated and grown on R2A and AIA, was flipped and seeded with *S. epidermidis* (left; R2A, dilution 10^−3^) and *P. putida* (right; AIA, dilution 10^−1^).

Although proving efficient, the spraying technique has two drawbacks: (i) for safety reasons, the procedure should be performed in a flow cabinet, which normally is not available in high school laboratories and (ii) positive bacteria are contaminated with tester bacteria by spraying and need purification. Therefore, as an alternative approach, reverse antibiosis was attempted by placing soil sample agar disks upside down in the lid of the Petri dish, and seeding tester bacteria on the bottom surface of the disks (now facing upwards) with a cotton swab. Validity of this approach was first tested with some purified actinobacteria identified in the MicroMundo project, that were seeded with *S. epidermidis* or *B. subtilis*, and with the two purified bacteria showing activity against *P. putida*, that were seeded with *P. putida* (Figure 2A). The flipping method was then applied to three MicroMundo soil samples (AB-02-06, AB-02-10, and AB-03-07) that contained ample antagonistic activity as identified by the high school students. At dilution 10^−6^, with only few colonies grown on the plates, about 50% of them already showed colonies surrounded by halos when probed with *S. epidermidis* (examples in Figure 2B), demonstrating the potential of the method. However, soil samples grown on R2A and AIA normally also contain quite a few soft and slimy colonies in addition to actinomycetes. After flipping the plate, such colonies get squeezed between agar and plate during the inoculation with tester bacteria, causing their spread and contamination of other colonies. This complicates screening and isolation of colonies on densely populated plates. Currently, tests in our laboratory are in progress to incorporate a simple heat treatment before plating to eliminate soft colonies from non-spore forming bacteria and allow enrichment of actinobacteria (Tiwari and Gupta, 2013). A test with applying heat (60°C for 20–60 min) to soil samples was found to reduce the number of colonies on the plates by about 90%. With a soil sample (AB-03-08) that had not yielded positive bacteria in the MicroMundo project, screening the heat-treated sample with the flipping method identified colonies with activity against *S. epidermidis*, as well as *P. putida* (Figure 2C). Thus, the pre-treatment makes the flipping technique suitable for screening, including identification of more rare activity against Gram-negative bacteria on densely populated plates. We, therefore, believe that this will become the preferred method for implementation in the educational MicroMundo program, as no special equipment or skills are required.

In conclusion, we have developed two reverse antibiosis approaches for targeted screening of actinobacteria that are easily applicable to identify antagonistic activity against Gram-negative as well as Gram-positive bacteria from soil.

## Data Availability Statement

DNA sequences presented in this study can be found in the online repository GenBank under accession numbers MT992770–MT992784. Results of MicroMundo Albacete 2020 are detailed in Supplementary Material.

## Ethics Statement

Ethical review and approval was not required for the human participants in accordance with the local legislation and institutional requirements. Written informed consent to participate in this study was provided by the participants’ legal guardian/next of kin.

## Author Contributions

PG, PC-C, and DM designed the study. PG and MA wrote the paper. PG, MA, and DM performed the experiments. All authors contributed to the article and approved the submitted version.

### Conflict of Interest

The authors declare that the research was conducted in the absence of any commercial or financial relationships that could be construed as a potential conflict of interest.
