# Supplementary material for: MicroMundo Upside Down: Targeted Searching for Antibiotics-Producing Bacteria From Soil With Reverse Antibiosis Approaches
Source: Front Microbiol. 2020 Oct 15;11:577550. doi: 10.3389/fmicb.2020.577550 (PMC7643607; doi:10.3389/fmicb.2020.577550)
Supplement: Supplementary file 1 [file Table_1.pdf]

SUPPLEMENTARY TABLE S1. Antibiotic-producing bacteria isolated in MicroMundo Albacete 2020.

Student pair & colony nr.	Soil sample coordinates (Dec. degrees)	Size of halo (mm)[a]		NCBI-Blast result[c]	Antibiotic produced by mentioned species	PMID
		Bs[b]	Se			
IES Tomás Navarro Tomás (AB-02); R2A medium						
03-04	38.974872,-1.851495	12	11	97.6% id. with *S. chartreusis* strain NBRC 12753 and *S. resistomycificus* NBRC 12814	Tunicamycin<br>Calcimycin	29463727<br>21173184
04-10	38.964652,-1.865985	10	13	99.7% id. with *S. africanus* SAI2BX2		
05-02	39.1133,-1.8394	no halo	10	100% id. with *S. rishiriensis* 5W3 and XFB-BL and *S. lavendulae* subsp. *lavendulae* DSM40713	Lactonamycin<br>Auricin	10348042<br>29496832
06-02	39.1132686,-1.8396792	14	20	n.d.		
06-03		14	20	n.d.		
06-05		15	20	n.d.		
06-06		14	20	n.d.		
06-07		15	20	98.7% id. with *S. ederensis* NBRC 15410	Factumycin<br>Tetrangomycin	29705162
06-08		15	20	Identical to AB-02-06-07		
08-03	38.987984,-1.902684	8	9	99.9% id. with e.g *S. globisporus* KCTC 9026 and *S. griseinus* NBRC 12869	Lidamycin	26853480
09-02	38.806001,-1.736804	no halo	16	99.64% id. with *S. turgidiscabies* strain 151-HR5	Antimycin A	18070040
10-03	388079,-1.17472	11	15	n.d.		
10-05		12	14	n.d.		
10-07		12	16	97.5% id. with *S. tauricus* strains	Taurimycin	6954874
10-09		11	14	n.d.		
IES Andrés de Vandelvira (AB-03); AIA medium						
01-01	39.1166308,-1.8368663	11	10	100% id. with e.g. *S. badius* and *S. rubiginosohelvolus* strains	Rubomycin	1225178
02-07	39.114728,-1.838520	9	no halo	99.1% id. with *S. viridochromogenes* subsp. *komabensis* JZY4-51S	Phosphinotricin	24498397
03-06	39.3635,-1.7116	10	7	99.9% id. with e.g *S. olivoviridis* and *S. mutomycini* strains	Thioviridamide	23995943
05-02	39.0676727,-1.7449020	11	no halo	99.9% id. with *S. netropsis* strains	Distamycin polyene	25415678<br>24935520
06-06	39.2411873,1.-9196721	7.5	no halo	n.d.		
07-01	39.2993571,2.0681035	11	13	99.9% id. with *S. argenteolus* strains	Carbapenem	24420617
07-06		11	11	n.d.		
07-07		8	11	n.d.		
07-08		8	12	n.d.		
10-07	39.004,-1.8775	8	12	99.9% id. with e.g *S. argenteolus* and *S. cinereorectus* strains		

[a]Size of agar discs without halo is 6 mm.
[b]Bs, *Bacillus subtilis*; Se, *Staphylococcus epidermidis*.
[c]Based on 16S rDNA sequences.
